# Supplementary material for: Reduction in Allergenicity and Induction of Oral Tolerance of Glycated Tropomyosin from Crab
Source: Molecules. 2022 Mar 21;27(6):2027. doi: 10.3390/molecules27062027 (PMC8950673; doi:10.3390/molecules27062027)
Supplement: Supplementary file 1 [file molecules-27-02027-s001.zip › molecules-1640301-supplementary.pdf]

Table S1 Specific IgE Levels and Symptoms of the Crab-Sensitive Patients

No.	Serum No.	Age	Gender	Crab specific IgE (kUA/L)	Specific IgE of TM from crab (OD 450 nm)	Symptoms
1	B119847	17	F	3.69	0.12	Cough
2	A281913	55	F	15.50	0.16	Eczema
3	B159030	8	M	24	0.14	Allergic purpura
4	B134851	1	F	6.47	0.15	Systemic lupus erthematosus
5	A819807	31	M	5.36	0.14	Eczema
6	B156635	12	M	2.69	0.12	Eczema
7	B134016	29	M	11.20	0.17	Cough
8	B450961	48	F	9.81	0.13	Lungs infected
9	B127123	67	M	0.15	0.07	-
10	A820983	23	F	0.09	0.06	-

Table S2 Prediction results of MHC-II binding area for mouse H-2 T cell epitopes from TM

No.	Allele	Start	End	Peptide	Adjusted rank
1	H2-IAd	1	13	MDAIKKKMQAMKL	22.38
2	H2-IAd	4	20	IKKKMQAMKLEKDNAMD	24.19
3	H2-IAd	55	69	NELDQAQEQLSAANT	22.2
4	H2-IAd	60	74	AQEQLSAANTKLDEK	27
5	H2-IAd	100	114	ERSEERLNTATTKLA	30
6	H2-IAd	111	127	TKLAEASQAADESERMR	28.87
7	H2-IAd	124	138	ERMRKVLENRSLSDE	25.5
8	H2-IAd	145	159	ENQLKEARFLAEEAD	29.5
9	H2-IAd	160	173	KYDEVARKLAMVE	24.95
10	H2-IAd	168	182	KLAMVEADLERAEER	26.5
11	H2-IAd	217	231	REETYKEQIKTLANK	25.95
12	H2-IAb	231	247	KLKAAEARAEFAERSVQ	28.96
13	H2-IEd	236	250	EARAEFAERSVQKLQ	14.5

Table S3 Prediction results of MHC-II binding for human HLA T cells epitopes from TM

No.	Allele	Start	End	Peptide	Adjusted rank
1	HLA-DRB4*01:01	1	16	MDAIKKKMQAMKLEKD	2.89
2	HLA-DRB4*01:01	2	16	DAIKKKMQAMKLEKD	2.4
3	HLA-DRB4*01:01	2	17	DAIKKKMQAMKLEKDN	2.42
4	HLA-DRB4*01:01	3	17	AIKKKMQAMKLEKDN	1.8
5	HLA-DRB4*01:01	3	18	AIKKKMQAMKLEKDNA	1.85
6	HLA-DRB4*01:01	3	16	AIKKKMQAMKLEKD	2.15
7	HLA-DRB4*01:01	3	19	AIKKKMQAMKLEKDNAM	2.9
8	HLA-DRB4*01:01	4	18	IKKKMQAMKLEKDNA	1.7
9	HLA-DRB4*01:01	4	17	IKKKMQAMKLEKDN	1.94
10	HLA-DRB4*01:01	4	19	IKKKMQAMKLEKDNAM	1.96
11	HLA-DRB4*01:01	4	16	IKKKMQAMKLEKD	2.81
12	HLA-DRB4*01:01	5	19	KKKMQAMKLEKDNAM	2.1
13	HLA-DRB4*01:01	5	18	KKKMQAMKLEKDNA	2.15
14	HLA-DRB4*01:01	5	20	KKKMQAMKLEKDNAMD	2.54
15	HLA-DRB1*03:01	8	22	MQAMKLEKDNAMDRA	2.6
16	HLA-DRB1*03:01	9	22	QAMKLEKDNAMDRA	1.83
17	HLA-DRB1*03:01	9	23	QAMKLEKDNAMDRAD	2.4
18	HLA-DRB1*03:01	10	23	AMKLEKDNAMDRAD	1.94
19	HLA-DRB1*03:01	10	22	AMKLEKDNAMDRA	2.03
20	HLA-DRB1*03:01	10	24	AMKLEKDNAMDRADT	2.6
21	HLA-DRB1*11:01	81	95	AEGEVAALNRRIQLL	2.7
22	HLA-DRB1*11:01	82	95	EGEVAALNRRIQLL	2.05
23	HLA-DRB1*11:01	82	96	EGEVAALNRRIQLLE	2.3
24	HLA-DRB1*11:01	82	94	EGEVAALNRRIQL	2.81
25	HLA-DRB1*11:01	83	96	GEVAALNRRIQLLE	1.83

26	HLA-DRB1*11:01	83	95	GEVAALNRRIQLL	2.03
27	HLA-DRB1*11:01	83	97	GEVAALNRRIQLLEE	2.1
28	HLA-DRB1*11:01	83	98	GEVAALNRRIQLLEED	2.89
29	HLA-DQA1*05:01/DQB1*02:01	86	100	AALNRRIQLLEEDLE	2.5
30	HLA-DQA1*05:01/DQB1*02:01	87	100	ALNRRIQLLEEDLE	2.15
31	HLA-DQA1*05:01/DQB1*02:01	88	100	LNRRIQLLEEDLE	2.49
32	HLA-DQA1*01:02/DQB1*06:02	102	117	SEERLNTATTKLAEAS	2.08
33	HLA-DQA1*01:02/DQB1*06:02	102	118	SEERLNTATTKLAEASQ	2.73
34	HLA-DQA1*01:02/DQB1*06:02	103	117	EERLNTATTKLAEAS	1.6
35	HLA-DQA1*01:02/DQB1*06:02	103	118	EERLNTATTKLAEASQ	1.62
36	HLA-DQA1*01:02/DQB1*06:02	103	119	EERLNTATTKLAEASQA	2.55
37	HLA-DQA1*01:02/DQB1*06:02	104	118	ERLNTATTKLAEASQ	1.3
38	HLA-DQA1*01:02/DQB1*06:02	104	117	ERLNTATTKLAEAS	1.4
39	HLA-DQA1*01:02/DQB1*06:02	104	119	ERLNTATTKLAEASQA	1.62
40	HLA-DQA1*01:02/DQB1*06:02	104	120	ERLNTATTKLAEASQAA	2.73
41	HLA-DQA1*01:02/DQB1*06:02	105	118	RLNTATTKLAEASQ	1.4
42	HLA-DQA1*01:02/DQB1*06:02	105	119	RLNTATTKLAEASQA	1.4

Continuation Table S3

43	HLA-DQA1*01:02/DQB1*06:02	105	117	RLNTATTKLAEAS	1.72
44	HLA-DQA1*01:02/DQB1*06:02	105	120	RLNTATTKLAEASQAA	1.85
45	HLA-DQA1*01:02/DQB1*06:02	105	121	RLNTATTKLAEASQAAD	2.73
46	HLA-DQA1*01:02/DQB1*06:02	106	121	LNTATTKLAEASQAAD	2.65
47	HLA-DQA1*01:02/DQB1*06:02	106	119	LNTATTKLAEASQA	2.69
48	HLA-DQA1*01:02/DQB1*06:02	106	118	LNTATTKLAEASQ	2.81
49	HLA-DQA1*03:01/DQB1*03:02	109	123	ATTKLAEASQAADES	2.1
50	HLA-DQA1*03:01/DQB1*03:02	109	124	ATTKLAEASQAADESE	2.54
51	HLA-DQA1*03:01/DQB1*03:02	110	124	TTKLAEASQAADESE	1.6
52	HLA-DQA1*03:01/DQB1*03:02	110	123	TTKLAEASQAADES	2.26

53	HLA-DQA1*03:01/DQB1*03:02	110	125	TTKLAEASQAADESER	2.65
54	HLA-DQA1*03:01/DQB1*03:02	111	124	TKLAEASQAADESE	1.72
55	HLA-DQA1*03:01/DQB1*03:02	111	125	TKLAEASQAADESER	2.4
56	HLA-DQA1*03:01/DQB1*03:02	111	126	TKLAEASQAADESERM	2.77
57	HLA-DQA1*03:01/DQB1*03:02	112	125	KLAEASQAADESER	2.8
58	HLA-DQA1*03:01/DQB1*03:02	112	126	KLAEASQAADESERM	2.8
59	HLA-DQA1*03:01/DQB1*03:02	112	124	KLAEASQAADESE	2.81
60	HLA-DQA1*04:01/DQB1*04:02	145	159	ENQLKEARFLAEEAD	2.9
61	HLA-DQA1*04:01/DQB1*04:02	146	159	NQLKEARFLAEEAD	2.37
62	HLA-DRB5*01:01	148	162	LKEARFLAEEADRKY	3
63	HLA-DRB5*01:01	149	162	KEARFLAEEADRKY	2.8
64	HLA-DRB5*01:01	149	163	KEARFLAEEADRKYD	2.9
65	HLA-DRB5*01:01	150	163	EARFLAEEADRKYD	2.58
66	HLA-DRB5*01:01	150	164	EARFLAEEADRKYDE	2.8
67	HLA-DRB5*01:01	151	164	ARFLAEEADRKYDE	2.69
68	HLA-DRB5*01:01	151	165	ARFLAEEADRKYDEV	2.8
69	HLA-DQA1*03:01/DQB1*03:02	163	177	DEVARKLAMVEADLE	2.9
70	HLA-DQA1*03:01/DQB1*03:02	164	177	EVARKLAMVEADLE	2.8
71	HLA-DQA1*03:01/DQB1*03:02	165	181	VARKLAMVEADLERAEE	1.6
72	HLA-DQA1*05:01/DQB1*02:01	165	179	VARKLAMVEADLERA	2.6
73	HLA-DQA1*03:01/DQB1*03:02	165	180	VARKLAMVEADLERAE	3
74	HLA-DQA1*03:01/DQB1*03:02	166	181	ARKLAMVEADLERAEE	0.99
75	HLA-DQA1*03:01/DQB1*03:02	166	182	ARKLAMVEADLERAEER	1.67
76	HLA-DQA1*05:01/DQB1*02:01	166	179	ARKLAMVEADLERA	2.15
77	HLA-DQA1*04:01/DQB1*04:02	166	181	ARKLAMVEADLERAEE	2.31
78	HLA-DQA1*05:01/DQB1*02:01	166	180	ARKLAMVEADLERAE	2.7
79	HLA-DQA1*05:01/DQB1*02:01	166	178	ARKLAMVEADLER	2.96
80	HLA-DQA1*03:01/DQB1*03:02	167	181	RKLAMVEADLERAEE	0.92

81	HLA-DQA1*03:01/DQB1*03:02	167	182	RKLAMVEADLERAEER	0.99
82	HLA-DQA1*03:01/DQB1*03:02	167	183	RKLAMVEADLERAEERA	1.55
83	HLA-DQA1*04:01/DQB1*04:02	167	181	RKLAMVEADLERAEE	1.6
84	HLA-DQA1*04:01/DQB1*04:02	167	182	RKLAMVEADLERAEER	2.31
85	HLA-DQA1*05:01/DQB1*02:01	167	181	RKLAMVEADLERAEE	2.6

Continuation Table S3

86	HLA-DQA1*03:01/DQB1*03:02	168	181	KLAMVEADLERAEE	1.29
87	HLA-DQA1*03:01/DQB1*03:02	168	182	KLAMVEADLERAEER	1.4
88	HLA-DQA1*04:01/DQB1*04:02	168	181	KLAMVEADLERAEE	1.72
89	HLA-DQA1*03:01/DQB1*03:02	168	183	KLAMVEADLERAEERA	1.73
90	HLA-DQA1*04:01/DQB1*04:02	168	182	KLAMVEADLERAEER	2.1
91	HLA-DQA1*03:01/DQB1*03:02	168	184	KLAMVEADLERAEERAE	2.73
92	HLA-DQA1*03:01/DQB1*03:02	169	182	LAMVEADLERAEER	1.18
93	HLA-DQA1*03:01/DQB1*03:02	169	181	LAMVEADLERAEE	1.29
94	HLA-DQA1*03:01/DQB1*03:02	169	183	LAMVEADLERAEERA	1.3
95	HLA-DQA1*03:01/DQB1*03:02	169	184	LAMVEADLERAEERAE	1.62
96	HLA-DQA1*04:01/DQB1*04:02	169	182	LAMVEADLERAEER	1.83
97	HLA-DQA1*04:01/DQB1*04:02	169	181	LAMVEADLERAEE	1.87
98	HLA-DQA1*04:01/DQB1*04:02	169	183	LAMVEADLERAEERA	2.2
99	HLA-DQA1*04:01/DQB1*04:02	169	184	LAMVEADLERAEERAE	2.65
100	HLA-DQA1*03:01/DQB1*03:02	169	185	LAMVEADLERAEERAES	2.73
101	HLA-DQA1*03:01/DQB1*03:02	170	183	AMVEADLERAEERA	1.4
102	HLA-DQA1*03:01/DQB1*03:02	170	184	AMVEADLERAEERAE	1.5
103	HLA-DQA1*03:01/DQB1*03:02	170	182	AMVEADLERAEER	1.72
104	HLA-DQA1*03:01/DQB1*03:02	170	181	AMVEADLERAEE	1.78
105	HLA-DQA1*03:01/DQB1*03:02	170	185	AMVEADLERAEERAES	2.42
106	HLA-DQA1*03:01/DQB1*03:02	171	184	MVEADLERAEERAE	1.29
107	HLA-DQA1*03:01/DQB1*03:02	171	183	MVEADLERAEERA	1.47

108	HLA-DQA1*03:01/DQB1*03:02	171	185	MVEADLERAEERAES	1.5
109	HLA-DQA1*03:01/DQB1*03:02	171	182	MVEADLERAEER	1.78
110	HLA-DQA1*03:01/DQB1*03:02	171	186	MVEADLERAEERAESG	2.42
111	HLA-DQA1*03:01/DQB1*03:02	172	187	VEADLERAEERAESGE	2.65
112	HLA-DQA1*03:01/DQB1*03:02	182	196	RAESGESKIVELEEE	2.4
113	HLA-DQA1*03:01/DQB1*03:02	182	197	RAESGESKIVELEEEL	3
114	HLA-DQA1*05:01/DQB1*02:01	183	197	AESGESKIVELEEEL	2
115	HLA-DQA1*03:01/DQB1*03:02	183	196	AESGESKIVELEEE	2.05
116	HLA-DQA1*03:01/DQB1*03:02	183	197	AESGESKIVELEEEL	2.1
117	HLA-DQA1*05:01/DQB1*02:01	184	197	ESGESKIVELEEEL	1.62
118	HLA-DQA1*03:01/DQB1*03:02	184	197	ESGESKIVELEEEL	1.72
119	HLA-DQA1*05:01/DQB1*02:01	184	198	ESGESKIVELEEELR	2.2
120	HLA-DQA1*03:01/DQB1*03:02	184	196	ESGESKIVELEEE	2.49
121	HLA-DQA1*05:01/DQB1*02:01	184	199	ESGESKIVELEEELRV	2.89
122	HLA-DQA1*05:01/DQB1*02:01	185	198	SGESKIVELEEELR	1.83
123	HLA-DQA1*05:01/DQB1*02:01	185	199	SGESKIVELEEELRV	1.9
124	HLA-DQA1*05:01/DQB1*02:01	185	197	SGESKIVELEEEL	2.03
125	HLA-DQA1*03:01/DQB1*03:02	185	197	SGESKIVELEEEL	2.96
126	HLA-DQA1*05:01/DQB1*02:01	186	199	GESKIVELEEELRV	1.62
127	HLA-DQA1*05:01/DQB1*02:01	186	198	GESKIVELEEELR	2.34
128	HLA-DQA1*05:01/DQB1*02:01	186	200	GESKIVELEEELRVV	2.4

Continuation Table S3

129	HLA-DQA1*05:01/DQB1*02:01	186	197	GESKIVELEEEL	2.85
130	HLA-DQA1*05:01/DQB1*02:01	187	200	ESKIVELEEELRVV	2.26
131	HLA-DQA1*05:01/DQB1*02:01	187	199	ESKIVELEEELRV	2.34
132	HLA-DRB5*01:01	194	207	EEELRVVGNNLKSL	2.8
133	HLA-DRB5*01:01	195	208	EELRVVGNNLKSLE	2.8
134	HLA-DRB1*08:02	219	234	ETYKEQIKTLANKLKA	1.96

135	HLA-DRB5*01:01	219	234	ETYKEQIKTLANKLKA	1.96
136	HLA-DRB1*08:02	219	233	ETYKEQIKTLANKLK	2.6
137	HLA-DRB5*01:01	219	235	ETYKEQIKTLANKLKAA	2.73
138	HLA-DRB5*01:01	220	234	TYKEQIKTLANKLKA	1.1
139	HLA-DRB1*08:02	220	234	TYKEQIKTLANKLKA	1.2
140	HLA-DRB1*08:02	220	235	TYKEQIKTLANKLKAA	1.73
141	HLA-DRB5*01:01	220	235	TYKEQIKTLANKLKAA	1.73
142	HLA-DRB1*08:02	220	233	TYKEQIKTLANKLK	2.26
143	HLA-DRB5*01:01	220	233	TYKEQIKTLANKLK	2.58
144	HLA-DRB5*01:01	220	236	TYKEQIKTLANKLKAAE	2.73
145	HLA-DRB1*08:02	220	236	TYKEQIKTLANKLKAAE	2.9
146	HLA-DRB5*01:01	221	234	YKEQIKTLANKLKA	0.84
147	HLA-DRB1*08:02	221	235	YKEQIKTLANKLKAA	0.86
148	HLA-DRB5*01:01	221	235	YKEQIKTLANKLKAA	0.88
149	HLA-DRB1*08:02	221	234	YKEQIKTLANKLKA	1.18
150	HLA-DRB1*08:02	221	236	YKEQIKTLANKLKAAE	1.73
151	HLA-DRB5*01:01	221	236	YKEQIKTLANKLKAAE	1.73
152	HLA-DRB5*01:01	221	237	YKEQIKTLANKLKAAEA	2.73
153	HLA-DRB1*11:01	221	235	YKEQIKTLANKLKAA	2.8
154	HLA-DRB5*01:01	221	233	YKEQIKTLANKLK	2.81
155	HLA-DRB1*08:02	221	237	YKEQIKTLANKLKAAEA	2.9
156	HLA-DRB1*08:02	222	235	KEQIKTLANKLKAA	0.66
157	HLA-DRB5*01:01	222	235	KEQIKTLANKLKAA	0.78
158	HLA-DRB5*01:01	222	234	KEQIKTLANKLKA	0.84
159	HLA-DRB1*08:02	222	236	KEQIKTLANKLKAAE	0.84
160	HLA-DRB5*01:01	222	236	KEQIKTLANKLKAAE	0.88
161	HLA-DRB1*08:02	222	237	KEQIKTLANKLKAAEA	1.73
162	HLA-DRB5*01:01	222	237	KEQIKTLANKLKAAEA	1.73

163	HLA-DRB1*08:02	222	234	KEQIKTLANKLKA	1.87
164	HLA-DRB5*01:01	222	238	KEQIKTLANKLKAAEAR	2.73
165	HLA-DRB1*08:02	223	236	EQIKTLANKLKAAE	0.72
166	HLA-DRB1*08:02	223	235	EQIKTLANKLKAA	0.78
167	HLA-DRB5*01:01	223	236	EQIKTLANKLKAAE	0.85
168	HLA-DRB5*01:01	223	235	EQIKTLANKLKAA	0.87
169	HLA-DRB1*08:02	223	237	EQIKTLANKLKAAEA	0.9
170	HLA-DRB5*01:01	223	237	EQIKTLANKLKAAEA	1.1
171	HLA-DRB5*01:01	223	234	EQIKTLANKLKA	1.45

Continuation Table S3

172	HLA-DRB1*08:02	223	238	EQIKTLANKLKAAEAR	1.85
173	HLA-DRB5*01:01	223	238	EQIKTLANKLKAAEAR	1.85
174	HLA-DRB1*08:02	223	234	EQIKTLANKLKA	2.28
175	HLA-DRB1*11:01	223	237	EQIKTLANKLKAAEA	3
176	HLA-DRB1*08:02	224	239	QIKTLANKLKAAEARA	1.85
177	HLA-DRB5*01:01	224	239	QIKTLANKLKAAEARA	2.42
178	HLA-DQA1*03:01/DQB1*03:02	229	243	ANKLKAAEARAEFAE	2.7
179	HLA-DQA1*03:01/DQB1*03:02	229	244	ANKLKAAEARAEFAER	2.89
180	HLA-DQA1*03:01/DQB1*03:02	230	244	NKLKAAEARAEFAER	1.6
181	HLA-DQA1*03:01/DQB1*03:02	230	243	NKLKAAEARAEFAE	2.26
182	HLA-DQA1*03:01/DQB1*03:02	230	245	NKLKAAEARAEFAERS	2.89
183	HLA-DQA1*03:01/DQB1*03:02	231	244	KLKAAEARAEFAER	1.94
184	HLA-DQA1*03:01/DQB1*03:02	231	245	KLKAAEARAEFAERS	2.3
185	HLA-DQA1*03:01/DQB1*03:02	231	246	KLKAAEARAEFAERSV	2.89
186	HLA-DQA1*03:01/DQB1*03:02	232	245	LKAAEARAEFAERS	1.94
187	HLA-DQA1*03:01/DQB1*03:02	232	244	LKAAEARAEFAER	2.18
188	HLA-DQA1*03:01/DQB1*03:02	232	246	LKAAEARAEFAERSV	2.3

189	HLA-DQA1*03:01/DQB1*03:02	232	247	LKAAEARAEFAERSVQ	2.89

Table S4 Identification of glycation sites of TM

Modified amino acid	Peptide sequence	Δ Mass	Molecular formula	MV
K12	MQAMK(1)LEK	52	C2H4O	54
K15	LEK(1)DNAMDR	52	C2H4O	54
R21	DNAMDR(1)ADTLEQQNK	52	C2H4O	54
K30	ADTLEQQNK(1)EANLR	52	C2H4O	54
R35	EANLR(1)AEK(1)TEEEIR	110	C6H8O2	112
R44	TEEEIR(1)ATQK	52	C2H4O	54
K49	K(1)MQQVENELDQAQEQLSAANTK	148	C5H10O5	150
K70	MQQVENELDQAQEQLSAANTK(1)	14	CH3	15
K74	LDEK(1)EK(1)ALQNAEGEVAALNR(1)R(1)	144	C5H10N2O3	146
K76	EK(1)ALQNAEGEVAALNR	52	C2H4O	54
R90	ALQNAEGEVAALNR(1)R	68	C4H6O	70
R91	R(1)IQLLEEDLER	72	CH2CH2COOH	73
R101	RIQLLEEDLER(1)	14	CH3	15
R105	IQLLEEDLER(1)SEER(1)	52	C2H4O	54
K112	LNTATTK(1)LAEASQAADESER	79	C5H4O	80
R125	LAEASQAADESER(1)	14	CH3	15
R127	LAEASQAADESER(1)MR(1)	52	C2H4O	54
K128	K(1)VLENR(1)SLSDEER	52	C2H4O	54
R133	VLENR(1)SLSDEER(1)	52	C2H4O	54
R140	SLSDEER(1)	14	CH3	15
K149	MDALENQLK(1)	14	CH3	15
R152	EAR(1)FLAEEADR	58	CH2COOH	59
R160	FLAEEADR(1)	14	CH3	15
K161	FLAEEADR(1)K(1)	52	C2H4O	54
R167	KYDEVAR(1)K	52	C2H4O	54

K168	K(1)LAMVEADLER	14	CH3	15
R178	LAMVEADLER(1)	14	CH3	15
R182	AEER(1)AESGESKIVELEEELR	148	C5H10O5	150
K189	AESGESK(1)IVELEEELR	132	C5H10O5	150
R198	IVELEEELR(1)	14	CH3	15
K205	IVELEEELR(1)VVGNNLK(1)	52	C2H4O	54
K213	SLEVSEEK(1)	14	CH3	15
R217	ANQR(1)EETYK(1)EQIK	52	C2H4O	54
K222	ANQR(1)EETYK(1)EQIK	52	C2H4O	54
K226	EQIK(1)TLANK	52	C2H4O	54
K231	TLANK(0.995)LK	52	C2H4O	54
K233	LK(1)AAEAR	52	C2H4O	54
R238	AAEAR(1)AEFAER	52	C2H4O	54
R244	AEFAER(1)SVQK	52	C2H4O	54
K248	AEFAERSVQK(1)	52	C2H4O	54
K251	LQK(1)EVDRLEDELVNEK	52	C2H4O	54

Continuation Table S4

R255	EVDR(1)LEDELVNEKEK	68	C4H6O	70
K264	EVDRLEDELVNEK(1)EK	79	C5H4O	80

Figuer S1 BMDCs uptake of FITC-labeled TM and TM-G followed in time

(A) BMDCs uptake of FITC-labeled TM followed in time.

(B) BMDCs uptake of FITC-labeled TM-G followed in time.

Figuer S2. Identification of glycation sites of TM-G

(A-D) identification of the specific amino acids of TM modified by the Maillard reaction using LC-MS/MS. The fragmention spectrums were shown in the graph with the amino acid in the figure corresponding to A_{22}-R_{35}, K_{49}-K_{70}, R_{91}-R_{101} and E_{150}-R_{160} of TM-G.

Figuer S1

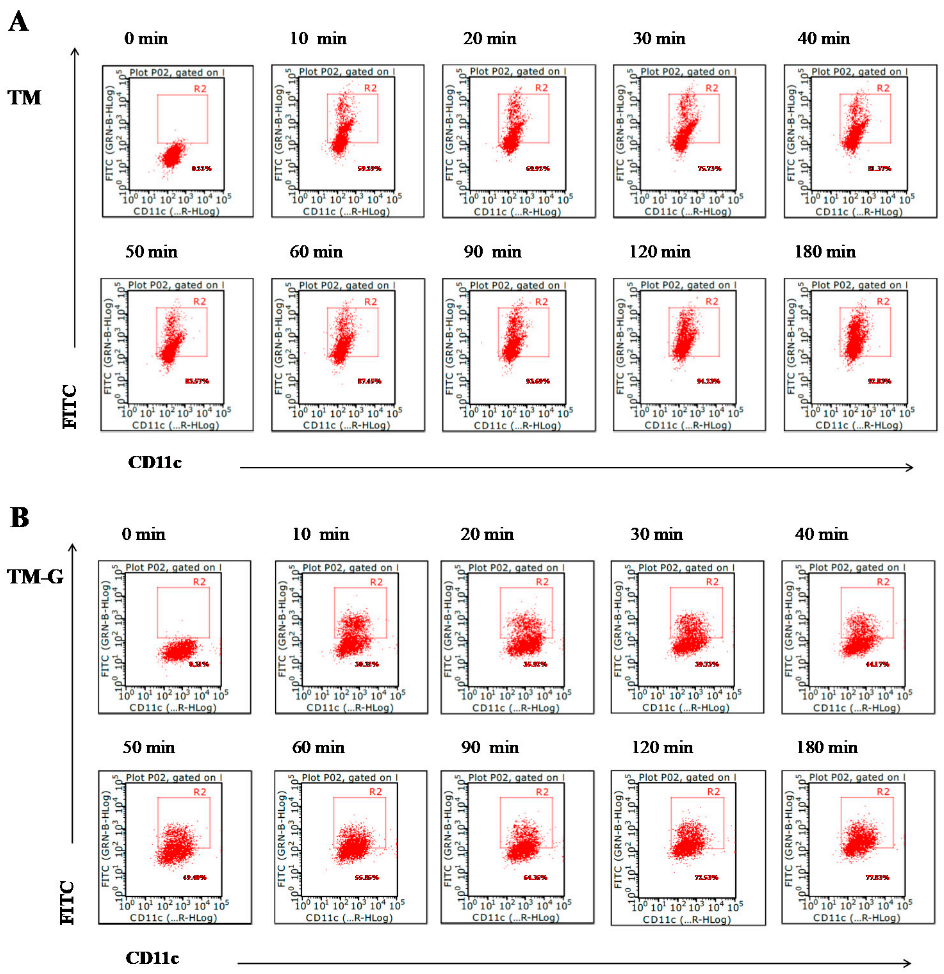
A
TM
0 min
10 min
20 min
30 min
40 min
50 min
60 min
90 min
120 min
180 min
Plot P02, gated on I
R2
FITC (GRN-B-HLog)
CD11c (...R-HLog)
FITC
CD11c
B
TM-G
0 min
10 min
20 min
30 min
40 min
50 min
60 min
90 min
120 min
180 min
FITC
CD11c

Figuer S2

A

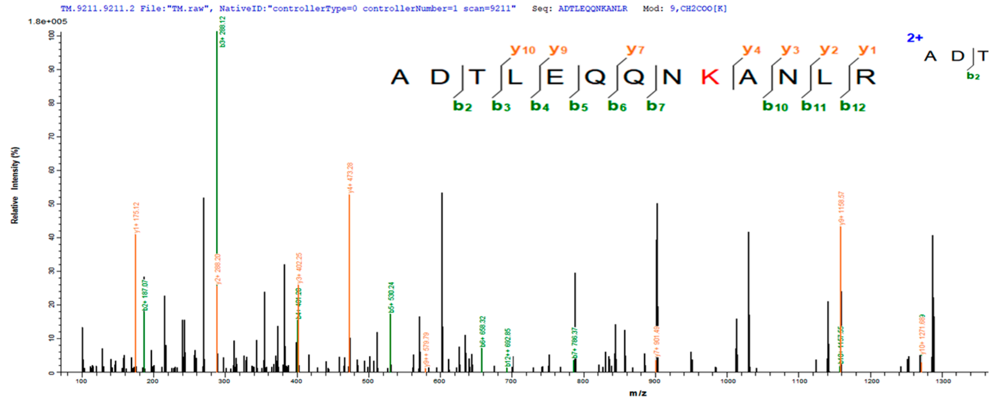

B

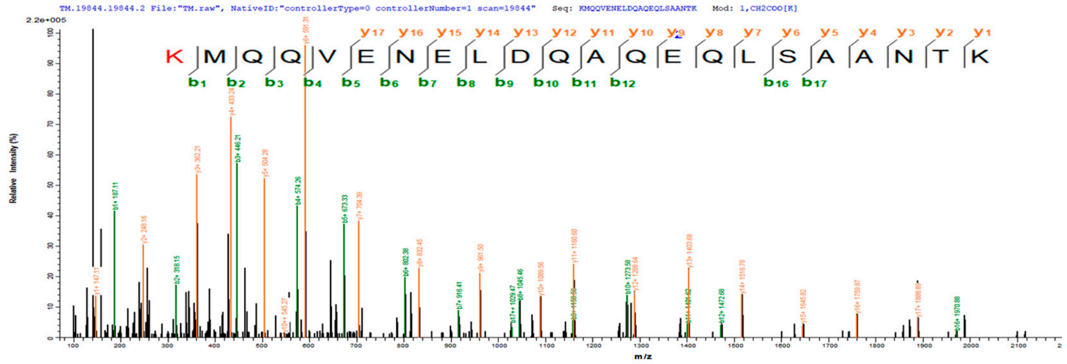

C

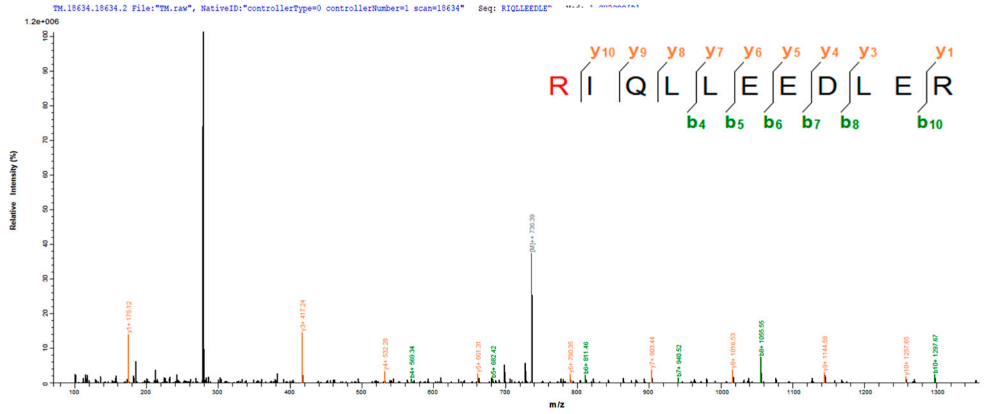

D

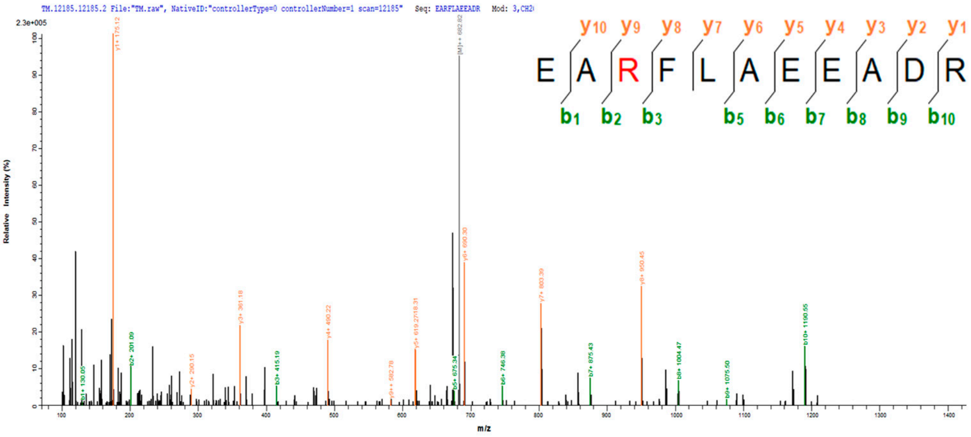
TM.12185.12185.2 File:"TM.raw", NativeID:"controllerType=0 controllerNumber=1 scan=12185" Seq: EARFLAEEADR Mod: 3,CH2
2.3e+005
Relative Intensity (%)
m/z
y1+ 175.12
[M]++ 682.82
y6+ 690.30
y8+ 950.45
y7+ 803.39
y3+ 361.18
y4+ 490.22
y5+ 619.27
b2+ 201.09
b10+ 1190.55
b3+ 415.19
b1+ 130.05
y2+ 290.15
y9++ 582.78
b5+ 675.34
b6+ 746.38
b7+ 875.43
b8+ 1004.47
b9+ 1075.50
y10 y9 y8 y7 y6 y5 y4 y3 y2 y1
E A R F L A E E A D R
b1 b2 b3 b5 b6 b7 b8 b9 b10
